# Chronic Kidney disease and cognitive frailty in aging: molecular crosstalk and clinical implications

**DOI:** 10.3389/fnagi.2026.1778574

**Published:** 2026-04-10

**Authors:** Ying Cao, Zihe Wang, Jiao Xue, Luo Wang, Wei Qin, Qianmei Sun, Jing Chang

**Affiliations:** 1 Department of Internal Medicine, Beijing Chao-yang Hospital, Capital Medical University, Beijing, China; 2 Department of General Medicine, Zhangjiawan Community Health Service Center of Tongzhou District, Beijing, China; 3 College of Computer Science, Beijing University of Technology, Beijing, China; 4 Department of Neurology, Beijing Chaoyang Hospital, Capital Medical University, Beijing, China; 5 Department of Nephrology, Beijing Chaoyang Hospital, Capital Medical University, Beijing, China

**Keywords:** artificial intelligence, chronic kidney disease, cognitive frailty, gut–kidney–brain axis, multi-omics, neuroimaging

## Abstract

**Background:**

Chronic kidney disease (CKD) and cognitive frailty frequently co-occur in older adults, compounding adverse health outcomes and placing substantial strain on healthcare systems.

**Summary:**

This review outlines the molecular pathways that link CKD to cognitive decline, including chronic inflammation, oxidative stress, vascular dysfunction, hormonal dysregulation, and interactions along the gut–kidney–brain axis. Recent advances in neuroimaging offer objective biomarkers of brain atrophy, white matter injury, and disrupted functional connectivity.

**Key messages:**

Multi-omics and single-cell technologies are uncovering cell-type-specific mechanisms and candidate biomarkers, paving the way for precision medicine approaches. Emerging strategies include integrated geriatric–nephrology care models and targeted interventions, such as SGLT2 inhibitors, IL-6 antagonists, microbiome modulation, and structured exercise programs. Bioengineering and artificial intelligence now enable the integration of multimodal data to support risk prediction, disease monitoring, and individualized therapeutic planning. Future priorities should focus on longitudinal cohort studies, interventional trials with cognitive endpoints, and the development of rigorously validated AI-driven predictive models. Effectively addressing CKD-related cognitive frailty will require translating molecular insights into clinical practice to mitigate vulnerability in aging populations.

## 1 Introduction

Chronic kidney disease (CKD) and cognitive frailty are increasingly prevalent among older adults, posing significant clinical and public health challenges (GBD 2021 Causes of Death Collaborators, 2024; Qiu et al., 2022). CKD affects approximately 9.1% of the global population, with higher prevalence rates in older age groups (Kidney Disease Improving Global Outcomes (KDIGO) CKD Work Group, 2024). Cognitive frailty, a condition defined by the co-occurrence of physical frailty and cognitive impairment, has emerged as a critical issue in geriatric care (Kelaiditi et al., 2013). CKD is associated with an elevated risk of cognitive impairment that extends beyond typical age-related cognitive decline and can be substantially worsened. In a national longitudinal cohort study of 17,708 participants followed for 8 years, the incidence of cognitive dysfunction was significantly higher in individuals with CKD than in those without, and its onset occurred 1.24 years earlier (Shang et al., 2024). The coexistence of CKD and cognitive frailty in older adults amplifies adverse outcomes, including greater morbidity, higher mortality, and reduced quality of life. This dual burden also imposes considerable strain on healthcare systems, underscoring the need for integrated management strategies that address the complex interplay between these conditions (Chang et al., 2022; Kennard et al., 2024).

CKD is classified into five stages based on glomerular filtration rate (GFR), ranging from mild kidney damage in Stage 1 to kidney failure in Stage 5. The progression of CKD involves multifaceted pathophysiological processes, including systemic inflammation, oxidative stress, vascular dysfunction, and hormonal imbalances. These mechanisms not only drive renal deterioration but also exert systemic effects that can impair cognitive function (Tang et al., 2024; Viggiano et al., 2020). CKD-related disturbances may converge on shared pathways of brain aging: systemic inflammation can promote neuroinflammation, uremic toxins may disrupt the blood–brain barrier and induce neuronal injury, oxidative stress can compromise mitochondrial and synaptic function, and vascular aging may contribute to cerebral small vessel disease, all of which heighten susceptibility to cognitive frailty. Studies indicate that individuals with CKD may exhibit deficits particularly in executive, attentional, and language-related domains, while dialysis populations also show prominent executive dysfunction (Levassort et al., 2024; Olejnik et al., 2024). Cognitive frailty is a geriatric syndrome characterized by the coexistence of physical frailty and cognitive impairment, which together increase vulnerability to adverse health outcomes. Unlike mild cognitive impairment (MCI), cognitive frailty explicitly incorporates both cognitive decline and physical frailty, reflecting a broader state of multisystem vulnerability with distinct clinical implications. In contrast to dementia, cognitive frailty is defined in the absence of dementia and is considered potentially reversible or modifiable, underscoring the critical importance of early detection and timely multidomain intervention (Kelaiditi et al., 2013; Nader et al., 2023).

**FIGURE 1:**
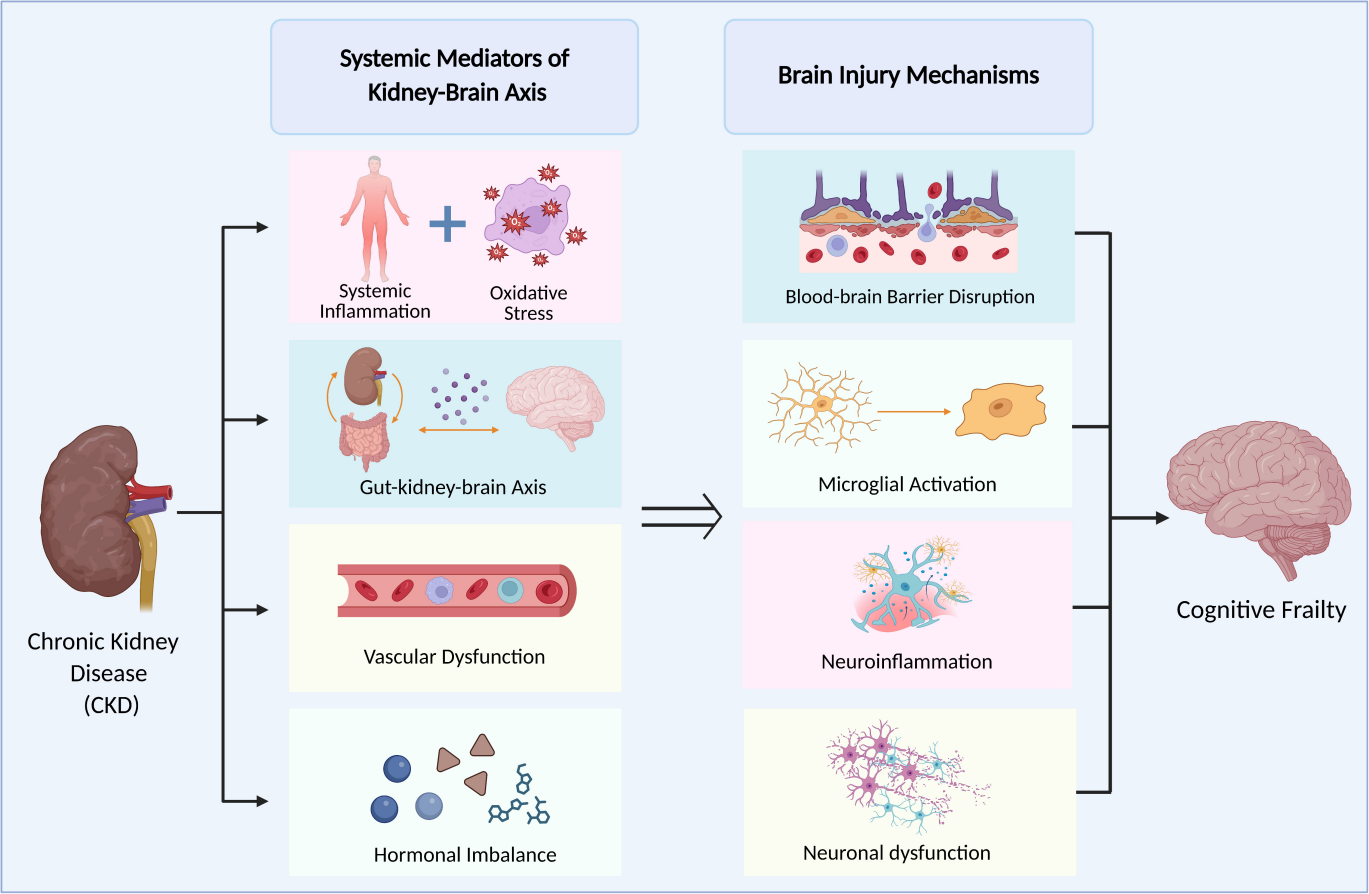
Conceptual framework of the kidney–brain axis linking CKD to cognitive frailty. CKD may contribute to cognitive frailty through multiple interconnected systemic mediators, including systemic inflammation, oxidative stress, gut–kidney–brain interactions, vascular dysfunction, and hormonal imbalance. These factors are not independent; rather, they may converge on shared brain injury pathways, such as blood–brain barrier disruption, microglial activation, neuroinflammation, and neuronal dysfunction. Together, these processes form a conceptual framework for understanding how kidney dysfunction may drive brain aging and increase susceptibility to cognitive frailty.

Despite the well-established association between CKD and cognitive frailty, the underlying molecular mechanisms remain poorly understood. Most current research has examined the effects of CKD and cognitive impairment in isolation, with limited attention to their combined impact or the potential for targeted interventions. Furthermore, many existing studies lack comprehensive integration of recent advances in molecular biology and geriatric medicine (Kennard et al., 2024). This review addresses these gaps by synthesizing the latest evidence on molecular crosstalk between CKD and cognitive frailty, highlighting emerging research priorities, and evaluating innovative approaches to clinical management. By integrating contemporary scientific insights, the review aims to deepen understanding of CKD-related cognitive frailty and support the development of effective, evidence-based interventions for older adults (Figure 1). These systemic alterations may disrupt the blood–brain barrier, activate microglia, and trigger neuroinflammation and neuronal dysfunction, thereby accelerating brain aging and contributing to cognitive frailty. Figure 1 provides a conceptual overview of the kidney–brain axis, illustrating how CKD-related systemic disturbances converge on shared pathways of brain injury that may underlie cognitive frailty.

This narrative review drew on literature retrieved from PubMed, Web of Science, and Scopus using a combination of free-text keywords and Medical Subject Headings (MeSH) terms. The search, conducted through November 2025, focused on chronic kidney disease, cognitive frailty, cognitive impairment, aging, molecular mechanisms, biomarkers, and emerging diagnostic or therapeutic strategies. Articles were selected based on their translational and clinical relevance to the kidney–brain axis and CKD-associated cognitive frailty.

## 2 Key molecular pathways

CKD and cognitive frailty are highly prevalent among older adults and frequently coexist, each accelerating the progression of the other. This review examines the molecular mechanisms linking CKD to cognitive decline, with a focus on recent advances in understanding inflammation, oxidative stress, the gut–kidney–brain axis, vascular dysfunction, hormonal dysregulation, and the application of multi-omics and single-cell technologies.

### 2.1 Inflammation and oxidative stress

Chronic low-grade inflammation, marked by persistently elevated inflammatory mediators, is a hallmark of CKD and a key driver of its progression. This state reflects an imbalance between pro-inflammatory and anti-inflammatory factors, which can promote neuroinflammation and contribute to cognitive decline (Chagas et al., 2025; Kadatane et al., 2023). Elevated levels of pro-inflammatory cytokines, such as IL-6 and TNF-α, are linked to CKD-related neurovascular injury and cognitive impairment (Bolignano et al., 2024). These cytokines first compromise the integrity of the blood–brain barrier (BBB), cross into the central nervous system, and subsequently activate microglia, thereby fueling neurodegenerative processes (Viggiano et al., 2020; Zimmermann et al., 2024). Oxidative stress, driven by excessive reactive oxygen species (ROS), further amplifies neuronal damage (Jurcau et al., 2024). In CKD, weakened antioxidant defenses lead to ROS accumulation, causing lipid peroxidation, DNA damage, and mitochondrial dysfunction (Ho and Shirakawa, 2022). Recent studies using single-cell RNA sequencing have identified distinct inflammatory cell populations in CKD models, offering new insights into cell-type-specific roles in disease progression (Bhayana et al., 2025).

### 2.2 Gut-kidney-brain axis

The gut microbiome, comprising more than 100 trillion bacteria, plays a critical role in maintaining metabolic and immune homeostasis. Disruption of microbial balance can trigger inflammation and metabolic disturbances that contribute to progressive declines in both kidney function and cognitive performance (Bakinowska et al., 2024; Liang et al., 2022). In CKD, dysbiosis of the gut microbiota drives the production and accumulation of gut-derived uremic toxins, such as indoxyl sulfate and p-cresyl sulfate (Tsuji et al., 2024), which compromise the BBB and promote neuroinflammation (Faucher et al., 2023). These toxins also suppress astrocyte glycolysis, leading to apoptotic cell death, and may contribute to the vascular dysfunction observed in CKD. Collectively, these mechanisms are linked to CKD-related cognitive impairment (Jeong et al., 2024). Moreover, increased intestinal permeability enables endotoxins to enter systemic circulation, further amplifying inflammatory responses (Tsuji et al., 2024). Therapeutic strategies targeting the gut microbiota, including probiotics and prebiotics, have shown promise in mitigating these effects. Current evidence suggests that microbiome-targeted interventions may lower gut-derived uremic toxins and inflammatory burden in CKD, but direct improvement in cognition remains insufficiently established in CKD-specific studies, and most support remains mechanistic or exploratory rather than definitive clinical proof (Liu et al., 2023; Mitrović et al., 2023; Yan Q. et al., 2024).

### 2.3 Vascular dysfunction

CKD is strongly associated with atherosclerosis, and endothelial dysfunction is nearly universal in this condition (Inserra et al., 2021). In CKD, endothelial dysfunction promotes vascular wall inflammation and injury, while the persistent low-grade systemic inflammation further aggravates endothelial damage, accelerating atherosclerosis and contributing to cognitive impairment (Viggiano et al., 2020). Increased arterial stiffness, commonly assessed by pulse wave velocity, reduces cerebral perfusion and elevates the risk of white matter lesions (Álvarez-Bueno et al., 2024). Advanced neuroimaging studies have identified microvascular abnormalities in CKD patients, including reduced capillary density and cerebral microbleeds, both of which are associated with cognitive deficits. Clinical and experimental studies have identified covert vascular brain injury and cerebral microbleeds in CKD, while additional work suggests systemic microangiopathy and impaired angiogenesis may further contribute to cerebral vulnerability (Fang et al., 2023; Miwa and Toyoda, 2022; Prommer et al., 2018). These vascular changes may also contribute to cerebral small vessel disease, thereby providing an additional link between CKD-related vascular aging and cognitive frailty.

### 2.4 Hormonal dysregulation

Renal dysfunction disrupts hormonal homeostasis, with adverse consequences for cognitive health. The kidneys produce erythropoietin (EPO), which exhibits neuroprotective effects by promoting neuronal growth and shielding the nervous system from injury. EPO deficiency is common in chronic kidney disease and has been associated with cognitive decline (Bhushan et al., 2025; Rey et al., 2019). Similarly, reduced levels of active vitamin D in CKD may accelerate neurodegenerative processes and contribute to cognitive deficits (Liu et al., 2025c). Recent CKD cohort data further suggest that severe vitamin D deficiency may be associated with a higher risk of mild cognitive impairment, although causality remains uncertain (Li et al., 2025). Elevated parathyroid hormone (PTH) levels have also been linked to neurotoxicity and impaired cognition (Crepeau et al., 2023). Therapeutic approaches targeting these hormonal imbalances, such as EPO supplementation and vitamin D analogs, are under investigation as potential strategies to improve cognitive outcomes in patients with CKD (Barbieri et al., 2024).

### 2.5 Multi-omics and single-cell technologies

Advances in multi-omics and single-cell sequencing have deepened our understanding of CKD-related cognitive decline. Integrated analyses of genomics, transcriptomics, proteomics, and metabolomics have uncovered potential biomarkers and therapeutic targets. These approaches help delineate the molecular pathways that connect kidney dysfunction to brain injury, particularly those involving inflammation, oxidative stress, vascular dysfunction, and metabolic disturbances. Single-cell RNA sequencing has further revealed the heterogeneity of neuronal and glial cell populations affected by CKD, identifying specific cell types and signaling pathways implicated in neuroinflammation and neurodegeneration. Together, these technologies offer mechanistic insights into CKD-associated cognitive impairment and provide a foundation for biomarker discovery and pathway-targeted interventions (Si et al., 2024; Zimmermann et al., 2024; Zoccali et al., 2025).

## 3 Clinical implications of CKD-cognition crosstalk

CKD is often accompanied by cognitive impairment, presenting significant challenges in clinical assessment and management. Building on the molecular pathways discussed, this section explores how these mechanisms can inform clinical evaluation, care models, and intervention strategies for patients with CKD-associated cognitive frailty.

### 3.1 Assessment challenges

Evaluating cognitive function in CKD patients is complicated by various confounding factors, including demographic and psychosocial variability, pharmacological influences, sleep and depressive disorders, metabolic disturbances, uremic encephalopathy, dialysis, and kidney transplantation, all of which can significantly affect cognitive evaluation outcomes (Pépin et al., 2021). Traditional neuropsychological assessments may not adequately account for these confounders, leading to potential misdiagnosis or underestimation of cognitive impairment (Pépin et al., 2021; Wang et al., 2025). Furthermore, standard cognitive tests often lack sensitivity to the specific cognitive domains affected in CKD, such as executive function. These assessments can also be labor-intensive and costly, particularly in settings with limited staffing and scarce training resources (Chan F. H. F. et al., 2024). Recent work has expanded interest in blood-based neurodegeneration and inflammation-related biomarkers as adjuncts to assessment; however, these markers should currently be regarded as supportive research tools rather than stand-alone diagnostic tests in CKD (Dhana et al., 2025; Dittrich et al., 2023).

### 3.2 Role of magnetic resonance imaging in assessing cognitive frailty in CKD

Magnetic resonance imaging (MRI) is an important research and adjunctive tool for evaluating brain changes relevant to cognitive frailty in patients with CKD. Unlike traditional neuropsychological tests, which can be subjective, MRI provides objective, noninvasive visualization of structural and functional brain changes. For example, structural brain imaging in CKD patients not receiving kidney replacement therapy has revealed cerebral atrophy and reduced white and gray matter density (Steinbach and Harshman, 2022). Advanced MRI techniques, including structural MRI, diffusion tensor imaging (DTI), and functional MRI (fMRI), detect brain abnormalities such as white matter hyperintensities, cortical thinning, and microvascular alterations. DTI, an MRI modality that captures high-resolution images of the brain’s microstructural architecture, demonstrates disrupted white matter integrity (Lepping et al., 2021; Zhang et al., 2022), while fMRI reveals altered patterns of neuronal activation during cognitive tasks, reflecting cognitive impairment in CKD (Hu et al., 2024). MRI also offers insights into brain connectivity and the effects of CKD-related vascular dysfunction and neuroinflammation (Pépin et al., 2024). These imaging findings not only aid in diagnosing cognitive decline but also support monitoring of disease progression and assessment of treatment efficacy. Integrating MRI into clinical practice provides a comprehensive approach to assessing cognitive frailty, complementing other diagnostic tools and enhancing early detection, disease monitoring, and targeted interventions for patients with CKD (Pépin et al., 2024; Steinbach and Harshman, 2022).

### 3.3 Integrated care models

The complex interplay between CKD and its cognitive consequences calls for an integrated care approach that bridges nephrology and geriatrics to meet patients’ multifaceted needs. Incorporating a comprehensive geriatric assessment into the care of CKD patients can be effectively accomplished through collaboration among geriatricians, nephrologists, and nursing staff. This multidisciplinary strategy aims to improve early detection of cognitive impairment and support a holistic management plan. Such a plan includes, though is not limited to, medical interventions, nutritional therapy, and psychological support, thereby addressing the diverse clinical and functional needs of individuals with CKD (Kalantar-Zadeh et al., 2022; Nixon et al., 2021). Structured care frameworks, such as the Chronic Care Model, have demonstrated improved outcomes in chronic disease management by fostering proactive, patient-centered care (Voorend et al., 2024). In addition, emerging technologies like telemedicine and wearable health devices offer innovative opportunities for remote monitoring of cognitive function and disease progression, enhancing patient engagement and facilitating timely interventions (Bui et al., 2025; Ellis et al., 2024).

### 3.4 Personalized intervention strategies

Tailoring interventions to an individual’s molecular and clinical profile offers a promising strategy for mitigating cognitive decline in CKD (Holland et al., 2024; Kalantar-Zadeh et al., 2022). Anti-inflammatory strategies, including SGLT2 inhibitors and IL-6 pathway modulation, may reduce systemic inflammation and could indirectly benefit brain health, but direct cognitive benefit in CKD remains unproven because cognition has rarely been a primary endpoint in major trials (Shin et al., 2024; Wada et al., 2023). Likewise, modulation of the gut microbiota with probiotics or prebiotics is supported mainly by mechanistic rationale and early clinical studies rather than validated cognition-focused CKD trials (Tang et al., 2023; Zhu et al., 2021). In addition, physical exercise has shown potential to improve cognitive performance and overall quality of life in some CKD patients (Otobe et al., 2021). Nevertheless, the current evidence remains heterogeneous and is largely confined to mechanistic plausibility, preclinical data, or exploratory clinical findings rather than validated clinical applications. Critically, cognitive outcomes are seldom designated as primary endpoints in CKD trials, which limits definitive conclusions about their direct clinical benefit.

## 4 Future directions

**FIGURE 2:**
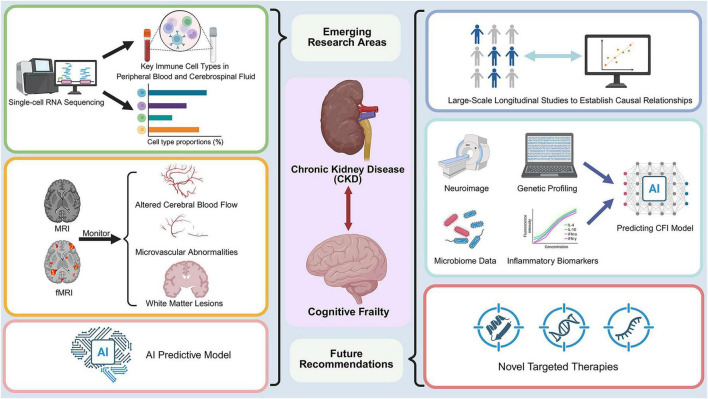
Future directions in CKD-related cognitive frailty: overview of emerging research areas and recommendations. This figure summarizes key priorities for future research in CKD-related cognitive frailty. Emerging research areas include single-cell and multi-omics approaches to characterize cellular heterogeneity, neuroimaging to monitor structural and functional brain changes, and AI-based predictive models that integrate multimodal data, including neuroimaging, genetic profiling, microbiome data, and inflammatory biomarkers. The figure also highlights future recommendations, such as large-scale longitudinal studies to establish causal relationships and the development of novel targeted therapies to improve prevention and management.

The complex molecular interplay between CKD and cognitive frailty in aging has become an area of growing scientific interest, yet several critical questions remain unanswered. Building on the mechanistic and clinical evidence reviewed above, this section outlines key priorities for future translational and clinical research. Although the biological pathways linking CKD to cognitive decline have received increasing attention, significant gaps persist in our understanding of the underlying mechanisms and in the development of effective therapeutic strategies. In the near term, research should prioritize biomarker validation and the integration of imaging with molecular data; in the midterm, the establishment of longitudinal cohorts and the development of integrated predictive models; and in the long term, the implementation of interventional trials and precision medicine approaches. This section highlights unresolved questions, emerging research areas, and offers recommendations to advance both the understanding and management of cognitive decline in patients with CKD (Figure 2). Figure 2 summarizes key emerging research directions and future recommendations for CKD-related cognitive frailty.

### 4.1 Unresolved questions

#### 4.1.1 Can the gut-kidney-brain axis become a novel therapeutic target?

The gut–kidney–brain axis represents a promising frontier in CKD-related cognitive frailty and has drawn increasing attention for its potential therapeutic implications (Alobaidi, 2025). Recent studies and emerging reviews suggest that gut microbiota dysbiosis may aggravate both kidney dysfunction and cognitive impairment, although the causal direction and effect size remain incompletely defined (Liu et al., 2025a). Pathways involving microbial-derived metabolites, such as indoxyl sulfate, and their capacity to affect brain function through inflammation, oxidative stress, immune modulation, and gut barrier interactions are gaining recognition (Tang et al., 2023). Nevertheless, it remains unclear whether targeting the gut microbiome or its metabolites could serve as a viable treatment strategy. For example, clinical trials evaluating probiotics or prebiotics as interventions for cognitive decline in CKD patients are still in early phases, and evidence regarding their efficacy in reducing uremic toxin production remains inconsistent and requires further investigation (Cooper et al., 2023). Given the complexity of this axis, future research should assess the feasibility and therapeutic potential of microbiome-targeted approaches. Modern sequencing technologies and advanced data analysis pipelines have been instrumental in advancing gut microbiome research, substantially deepening our understanding of the human intestinal microbial ecosystem and enabling targeted interventions for specific microbial communities. The integration of high-throughput sequencing with metabolomics now allows for the identification and relative quantification of diverse microbial species. Moreover, emerging tools such as high-throughput culturing, advanced microbial profiling, and integrative metabolomics are opening new avenues for disease prevention and therapeutic development (Song H. S. et al., 2024). Specifically, more rigorous clinical trials and well-designed animal models are needed to determine whether modulating the gut microbiome can improve cognitive outcomes in CKD patients. Equally important is investigating whether this axis might serve as a predictive biomarker for cognitive decline in CKD, thereby supporting earlier diagnosis and timely intervention.

#### 4.1.2 Can multi-omics approaches predict cognitive trajectories in CKD?

The integration of multi-omics, encompassing genomics, proteomics, transcriptomics, and metabolomics, has substantially advanced our understanding of CKD pathogenesis and enabled the identification of targeted molecular pathways. These advances open new avenues for developing innovative therapeutic strategies for kidney diseases (Hill et al., 2022; Zhou et al., 2023). Integrating these insights with current knowledge of the mechanisms driving CKD-related cognitive impairment holds considerable promise for elucidating the molecular basis of cognitive decline in this population. Multi-omics frameworks have been increasingly discussed in CKD-related multisystem complications, including sarcopenia, as potential tools for biomarker discovery and risk stratification (Chang et al., 2025). However, their potential to predict cognitive trajectories in CKD patients remains largely unexplored. It is still unclear whether combining multiple omics layers can uncover the biological underpinnings of cognitive decline and support the development of personalized treatment plans.

Recent advances in single-cell sequencing technologies now enable an unprecedented level of resolution in studying kidney–brain interactions at the cellular level. These techniques may reveal cell-specific alterations that contribute to cognitive dysfunction in CKD. For instance, a single-cell sequencing study demonstrated that CKD-associated cognitive impairment is linked to potassium efflux from microglia and neurons, offering novel insights for clinical prevention and treatment of CKD-related cognitive disorders (Zimmermann et al., 2024). Nevertheless, significant challenges remain in integrating these complex datasets, and further research is needed to validate the clinical utility of multi-omics approaches in predicting disease progression.

### 4.2 Emerging research areas

#### 4.2.1 Unveiling disease heterogeneity using multi-omics and single-cell technologies

Recent advances in multi-omics and single-cell sequencing now enable comprehensive mapping of the cellular and genetic landscape, facilitating the identification of cellular heterogeneity and intercellular interactions. These technologies have dramatically expanded our capacity to investigate molecular-level complexity in both cellular diversity and disease heterogeneity (Baysoy et al., 2023). They also allow a more precise understanding of how distinct cell populations in the kidney and brain respond to CKD and contribute to cognitive decline. For example, single-cell and related immune profiling studies in neurodegenerative disease have identified immune cell populations in the cerebrospinal fluid that may be relevant to cognitive disorders (Gate et al., 2020). In a separate study, researchers used single-cell RNA sequencing to construct a detailed cellular atlas of the prefrontal cortex in aging individuals, revealing specific glial and neuronal subsets associated with Alzheimer’s disease (Green et al., 2024). Although these single-cell findings primarily stem from studies of aging and neurodegenerative diseases rather than CKD-specific cohorts, they offer a valuable conceptual framework for understanding how cellular heterogeneity might also underlie CKD-associated cognitive frailty. In particular, such insights may help pinpoint vulnerable cell populations and molecular pathways relevant to risk stratification and therapeutic targeting in CKD patients. Here, we emphasize their future translational potential, not their general mechanistic role discussed earlier, in refining patient subgroup classification, identifying high-risk individuals, and informing more precise intervention strategies. These findings lay a cellular foundation for understanding cognitive decline and could guide the development of tailored therapeutic approaches.

Further exploration of how to integrate these discoveries into clinical practice is essential. Characterizing the molecular heterogeneity among CKD patients, including how genetic, epigenetic, and environmental factors shape disease progression, will aid in identifying those at greatest risk for cognitive decline (Vivante, 2024; Yan Y. et al., 2024). In this context, the translational value of multi-omics and single-cell approaches extends beyond mechanistic discovery to their potential for enabling more accurate subgroup identification and pathway-informed interventions in CKD-related cognitive frailty. This knowledge can ultimately support personalized treatment strategies that target the most relevant biological pathways.

#### 4.2.2 Neuroimaging in monitoring brain changes associated with CKD

Neuroimaging techniques are increasingly used to monitor structural and functional brain changes in patients with CKD. Recent studies have shown that CKD is associated with altered cerebral blood flow, microvascular abnormalities, and white matter lesions, all of which contribute to cognitive impairment. MRI and functional MRI (fMRI) have provided valuable insights into how the brain responds to CKD (Hu et al., 2024; Michna et al., 2020).

In the coming years, neuroimaging may play an increasingly important role in identifying early biomarkers of cognitive decline and evaluating therapeutic responses in CKD, although CKD-specific validation remains limited (Liu et al., 2025b; Song L. et al., 2024; Veitch et al., 2022). Recent multimodal MRI studies in CKD have strengthened the case for imaging markers linked to neurovascular coupling, functional connectivity, and stage-related cognitive decline (Liu et al., 2025b; Song L. et al., 2024). Moreover, integrating neuroimaging with peripheral biomarkers from blood or urine could improve early detection and deliver a more comprehensive assessment of brain health in CKD patients, potentially supporting more personalized treatment approaches (Andrade et al., 2025; Wang H. et al., 2023).

#### 4.2.3 The role of bioengineering and artificial intelligence in intervention design

The integration of bioengineering and artificial intelligence (AI) into the management of CKD-related cognitive decline opens new avenues for diagnosis and personalized care. In one study, researchers combined neuroimaging techniques to capture structural and functional information about brain networks and then applied an AI model to analyze this data and build a predictive model for cognitive impairment or frailty. This approach illustrates the synergistic potential of bioengineering, through neuroimaging, and AI in studying CKD-related cognitive decline (Wang Y. F. et al., 2023). Bioengineering innovations, such as wearable-based physiological monitoring combined with machine learning (Rykov et al., 2024), and emerging micro- or nanoscale delivery platforms with potential relevance for targeted therapeutics (Ahadian et al., 2020), may eventually contribute to more personalized management strategies. At the same time, AI algorithms are being developed to integrate and interpret large-scale datasets from multi-omics, neuroimaging, and electronic health records, uncovering patterns that can guide clinical decision-making. This strategy enhances the effective use of multimodal biomedical data and accelerates progress toward precision medicine (Graham et al., 2020; Tong et al., 2024).

AI also holds promise for designing more sophisticated, individualized interventions in engaging formats, for example, adaptive cognitive training programs (Chan A. T. C. et al., 2024) or personalized physical activity regimens that adjust in real time to a patient’s cognitive and physical status (Vandelanotte et al., 2023). Furthermore, AI models can help clinicians detect early-stage cognitive decline using non-invasive biomarkers, thereby reducing dependence on time-consuming and costly neuropsychological assessments (Almgren et al., 2023; Mohammadi et al., 2024).

### 4.3 Recommendations for future research

#### 4.3.1 Design of large-scale longitudinal studies to establish causal relationships

A major gap in current research is the lack of large-scale longitudinal studies capable of establishing a causal relationship between CKD and cognitive decline. Most existing evidence comes from cross-sectional or observational designs, which limit definitive conclusions about the directionality of this association (Chan F. H. F. et al., 2024; Levassort et al., 2024). Interpretation is further hindered by the substantial heterogeneity of CKD populations, particularly differences in CKD stage, dialysis modality, transplantation status, and comorbidities such as diabetes and hypertension. Future longitudinal studies should employ more refined stratification to clarify subgroup-specific risks. They should also prioritize biomarker-driven designs that integrate molecular and neuroimaging markers, with cognition specified as a primary outcome whenever feasible. Large, multicenter, longitudinal cohorts are urgently needed to track cognitive trajectories in CKD patients over time, identify modifiable risk factors, and determine how early-stage CKD influences cognitive decline. Recent cohort evidence supports an association between CKD burden and cognitive risk across diverse populations, but truly biomarker-integrated longitudinal datasets remain scarce (González et al., 2025; Shang et al., 2024; Tang et al., 2022). Although longitudinal CKD-specific datasets remain limited, AI and machine-learning approaches may help leverage complex multimodal data for prediction and risk stratification (Kale et al., 2024). Such studies should also account for CKD heterogeneity and incorporate multi-omics and neuroimaging data to capture the full spectrum of biological and clinical factors shaping cognitive function (Graham et al., 2020).

#### 4.3.2 Development of integrated diagnostic and predictive models

The complexity of CKD-related cognitive impairment calls for a comprehensive diagnostic approach that integrates multiple biomarkers and clinical indicators (Nader et al., 2023; Wang Y. F. et al., 2023). Future research should focus on developing integrated models that combine neuroimaging, genetic profiling, inflammatory biomarkers, and microbiome data to build robust diagnostic and predictive tools (Tong et al., 2024). Such models would not only support early detection but also help stratify the risk of cognitive decline in CKD patients. In addition, they could identify individuals most likely to benefit from specific interventions, such as anti-inflammatory therapies or microbiome modulation, enabling more targeted clinical management.

#### 4.3.3 Exploration of novel targeted therapies

Targeted therapies that address the mechanisms underlying CKD-related cognitive decline remain a critical but still early-stage research priority (Merlino et al., 2025). Recent studies have highlighted several promising targets, including inflammatory cytokines, oxidative stress pathways, and the gut microbiota (Zhu et al., 2025). Future clinical trials should prioritize evaluating interventions directed at these pathways, including anti-inflammatory strategies, microbiome-targeted approaches, and multidomain rehabilitation programs (Pépin et al., 2023; Shin et al., 2024; Tang et al., 2023; Wada et al., 2023; Zhu et al., 2021).

## 5 Conclusion

CKD-related cognitive frailty likely arises through a kidney–brain axis in which systemic inflammation, oxidative stress, vascular dysfunction, gut dysregulation, and hormonal imbalance converge to promote brain injury and cognitive decline. Neuroimaging, multi-omics approaches, and AI-based models hold promise for enabling earlier detection and more precise risk stratification. However, most candidate biomarkers and interventions remain insufficiently validated for cognition-specific clinical endpoints in CKD. Future research should prioritize longitudinal, biomarker-driven studies and the development of targeted interventions to improve clinical management of this complex condition.
